# Supplementary material for: Complexome profiling of the Chlamydomonas psb28 mutant reveals TEF5 as an early PSII assembly factor
Source: Plant Cell. 2025 Mar 17;37(6):koaf055. doi: 10.1093/plcell/koaf055 (PMC12164590; doi:10.1093/plcell/koaf055)
Supplement: koaf055_Supplementary_Data [file koaf055_supplementary_data.zip › psb28_interactive.zip!/plots/intensity/qualityPlots/peptides/_Cre10.g464050.t1.1.html]

### Cre10.g464050.t1.1

Trivial name:   
SeqCoverage [median]: 16.70  
Mass: 39.71 kDa  
  
 

| Sequence | MissedCleavage | Mass [Da] | wtIntensity | muIntensity | Score |
| --- | --- | --- | --- | --- | --- |
| IGEIAGIKLDR | 1 | 1183.69 | 0.00 | 6977.82 | 69.45 |
| ITPVGNVADLK | 0 | 1125.64 | 0.00 | 1145.42 | 52.58 |
| ITPVGNVADLKK | 1 | 1253.73 | 6816.98 | 10873.49 | 94.19 |
| QVEAEAR | 0 | 801.40 | 959.69 | 2939.50 | 96.49 |
| WVAAAEDEQGR | 0 | 1230.56 | 0.00 | 367.31 | 93.55 |
| YEVDLSDPR | 0 | 1092.51 | 0.00 | 506.15 | 101.64 |
|  |  |  | 7776.7 | 22809.7 |  |
